# Autologous tumor cells/bacillus Calmette-Guérin/formalin-based novel breast cancer vaccine induces an immune antitumor response

**DOI:** 10.18632/oncotarget.25044

**Published:** 2018-04-17

**Authors:** María José Godoy-Calderón, Víctor Salazar, Eglys González-Marcano, Ana Federica Convit

**Affiliations:** ^1^ Unidad Experimental de Inmunoterapia, Fundación Jacinto Convit, Caracas, Venezuela; ^2^ Jacinto Convit World Organization, Inc., Palo Alto, California, United States of America; ^3^ Servicio de Microscopía de Luz, Centro de Biofísica y Bioquímica, Instituto Venezolano de Investigaciones Científicas-IVIC, Caracas, Venezuela

**Keywords:** cancer immunotherapy, breast cancer, autologous tumor cells vaccine, BCG, formalin, Immunology

## Abstract

Autologous cancer cell vaccines represent a multivalent patient-specific treatment. Studies have demonstrated that these immunotherapies should be combined with immunomodulators to improve results. We tested in breast cancer the antitumor effects of a 200 µg autologous tumor cells homogenate combined with 0.0625 mg of bacillus Calmette-Guérin (BCG), and 0.02% formalin. We used a 4T1 murine model of BALB/c receiving four weekly injections of either this vaccine or control treatments. The control treatments were either Phosphate Buffer Saline, BCG treated with formalin, or the tumor cells homogenate plus BCG alone. We found that mice treated with the vaccine had the lowest tumor growth rate and mitosis percentage. The vaccinated group also showed a marked increase in infiltration of antitumor cells (natural killer, CD8^**+**^ T and CD4^+^ Th1 cells), as well as a decrease of myeloid-derived suppressor cells (MDSCs) and tumor-associated macrophages (TAMs). Additionally, we also observed a possible activation of the immune memory response as indicated by plasma cell tumor infiltration. Our results demonstrate that our proposed breast cancer vaccine induces a potent antitumor effect in 4T1 tumor-bearing mice. Its effectiveness, low cost and simple preparation method, makes it a promising treatment candidate for personalized breast cancer immunotherapy.

## INTRODUCTION

Breast cancer is the second most common cancer worldwide and one of the leading causes of malignancy-associated deaths among women [1]. Approximately 80% of patients receive adjuvant therapy such as chemotherapy, hormonal and/or radiotherapy, following tumor resection [2]. In an effort to combat tumor recurrence, immunotherapy has emerged as a therapeutic approach to attempt to overcome the immunosuppression in the tumor microenvironment. This approach leads to more effective tumor elimination by immune cells, while avoiding the side effects associated with chemo and radiotherapy toxicity [3].

Tumor cells alone have poor immunogenicity. Thus, many autologous tumor cells vaccine-based trials for breast cancer combine autologous tumor cells with additional antigens, cytokines or other immunomodulators [2]. The bacillus Calmette-Guérin (BCG) represents one of the most used adjuvants in immunotherapy regimens. Morales *et al.* in 1976 [4] reported a successful treatment of superficial bladder cancer with BCG. This immunotherapy is today FDA-approved as a standard treatment for this type of cancer [5]. BCG activates the immune system against tumors, triggering a Th1 immune response. For bladder cancer treatment, when BCG is instilled, cancer cells upregulate the expression of the major histocompatibility complex (MHC) class II and ICAM-1 and secrete various cytokines. BCG promotes dendritic cells (DCs) and recruits immune cells, initially granulocytes, followed by macrophages and lymphocytes. Toll-like Receptors (TLRs) participate in BCG recognition by urothelial cells and immune cells, secretion of proinflammatory cytokines and factors such as TNF-related apoptosis-inducing ligand (TRAIL). Activation of natural killer (NK) cells and secretion of TRAIL by polymorphonuclear cells have shown to lead to cytotoxicity of bladder cancer cells [6]. BCG has been used in combination with cyclophosphamide, irradiated autologous tumor cells, and 5-fluorouracil-Adriamycincyclophosphamide against different types of tumors, such as melanoma [7], colon carcinoma [8], and breast cancer [9] respectively, leading to improvements over the single agents.

BCG has also been used as an immune adjuvant in the treatment of infectious diseases such as leprosy and leishmaniasis, conditions that are thought to have specific immunological deficits at their core. BCG was an effective adjuvant in treating those diseases, particularly when modified with a dilute solution of formaldehyde [10–12]. Based on the success of these therapies, the parallels between the ineffective natural immune response to those infections among affected individuals, and the immunosuppressive qualities of cancer cells, an autologous tumor cells vaccine using this approach for the treatment of breast cancer was proposed [13, 14]. Later, an uncontrolled clinical study was described in advanced stage breast cancer patients, using autologous tumor cells combined with BCG and diluted formalin alone (for those women refusing further standard treatment), or in addition to standard chemotherapy/radiotherapy, demonstrating the feasibility and safety of this immunotherapy [15].

The current report describes the results of a preclinical study and provides mechanistic data for this therapeutic autologous tumor cells homogenate combined with BCG and diluted formalin, henceforth referred to as “the vaccine”, in a mouse 4T1 breast cancer model. This vaccine induced an immune antitumor response, thus supporting the proposed vaccine as a viable personalized immunotherapy.

## RESULTS

### 4T1 tumor morphological changes induced by each of the 4 treatment arms: PBS vehicle only (G1), BCG/formalin (G2), autologous tumor cells/BCG (G3), and autologous tumor cells/BCG/formalin (G4)

**Figure 1:**
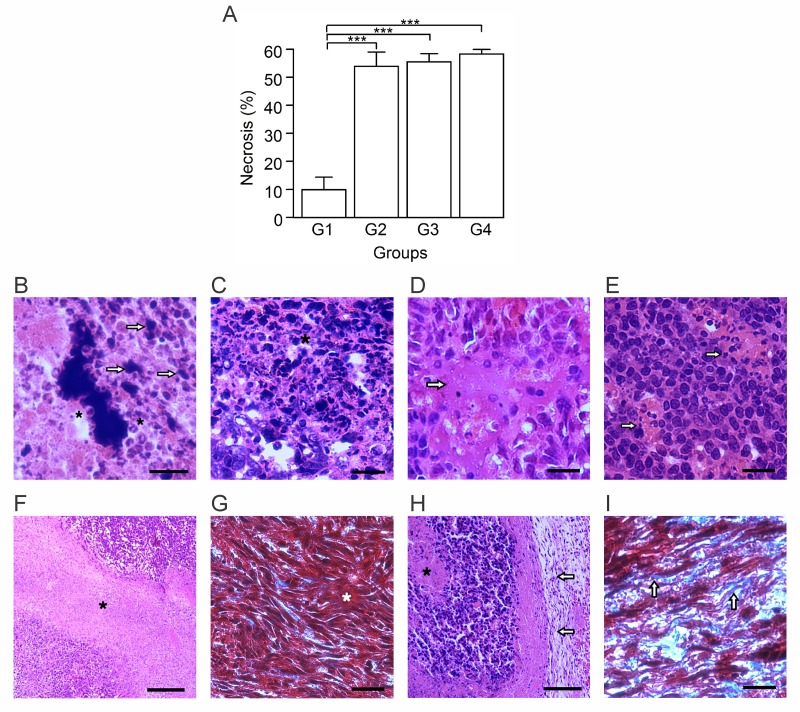
Autologous tumor cells/BCG/formalin vaccine induces morphological changes and extensive necrosis in 4T1 tumor Tumor slides obtained from each mice group were stained with H&E or Gomori´s trichome. (**A**) Tumor necrosis percentage was determined, resulting in higher values in G2, G3 and G4, with respect to G1. The data is shown as the mean percentage ± SEM of five mice per group (^***^p ≤ 0.001). (**B**) As an indicative of a long-term process, G4 showed necrotic zones surrounded by infiltrating leukocytes (asterisks) with lipofucsin bodies (arrows). (**C**) G4 presented necrotic foci with leukocytes infiltration (asterisk), condition that was also seen in G2 and G3. (**D** and **E**) G3 (D) and G4 (E), presented lytic necrosis (arrows in D) with minimal mononuclear infiltration (arrows in E). (**F** and **G**) G3 showed necrotic zones (asterisk in F) with absence of a delimited border, and fibroblast-like cells with scarce collagen fibers (asterisk in G). These histologic characteristics were also seen in G1. (**H** and **I**) G4 presented a connective tissue sheath (arrows in H), surrounding necrotic zones (asterisk in H), with abundant collagen fibers (arrows in I). Some slides were stained with H&E (B, C, D, E, F and H). The others with Gomori´s trichome (G and I) were the nuclei are stained in black; cytoplasm, keratin, muscle fibers in red; collagen and mucus in green or blue. Scale bar, 20 μm (B); 10 μm (C, D, E, G and I); 40 μm (F and H). *n* = 5 mice per group. Tukey’s post hoc significance (^***^p ≤ 0.001).

To determine the treatment effects over the tumor morphology, we performed a histological examination of tumor sections for each of the treatment arms (Table 1). Tumors corresponding to G1 were enveloped by sheets of dense connective tissue, and infiltrated by mononuclear and polymorphonuclear cells. In all treatment arms, the proliferative zone of the tumor, referred to as zone 1 (Z1), was composed of cells in constant mitosis with large nuclei and scarce cytoplasm. Next to Z1, there was presence of large lymphatic vessels, blood vessels, and tumor cells that constitute what is referred to as zone 2 (Z2). All active treatments induced high necrosis levels relative to G1 (*p* ≤ 0.05) (Figure 1A). The necrosis appears to begin in the tumor core and extend to the periphery, generating necrotic zones surrounded by infiltrating leukocytes with lipofucsin bodies, indicating a long-standing process (Figure 1B). Particular patterns of necrosis were found in each group: G1 showed a coagulative necrosis located in the core area that was poorly infiltrated, while G2, G3, and G4 presented necrotic foci with eosinophilic material, neutrophilic infiltration and cellular debris (Figure 1C). Particularly, G3 and G4 showed lytic necrosis with eosinophilic material, lysed cells, and minimal mononuclear cell infiltration (Figure 1D and 1E). Fibroblasts and collagen were detected mainly in G2 and G4. In G1 and G3 collagen fibers were poorly organized (Figure 1F and 1G), while in G4 they were located in connective tissue sheets surrounding necrotic zones with a more organized and developed structure (Figure 1H and 1I). Additionally, in the treated groups the BCG bacilli were detected in the tumor stroma. In G3, a treatment without formalin, the BCG was located inside the phagocytes. On the contrary, in G2 and G4, treatments with diluted formalin as a component, BCG was found in the extracellular space (data not shown).

**Table 1:** Treatments administered to each mice group

Group	Treatment
**G1**	PBS
**G2**	BCG (0.0625 mg/mouse) plus formaldehyde (0.02%/mouse)
**G3**	Autologous tumor cells homogenate (200 µg/mouse) plus BCG (0.0625 mg/mouse)
**G4**	Autologous tumor cells homogenate (200 µg/mouse) plus BCG (0.0625 mg/mouse) plus formaldehyde (0.02%/mouse) vaccine

### Autologous tumor cells/BCG/formalin vaccine induces marked inhibition of tumor growth and tumor cell proliferation

**Figure 2:**
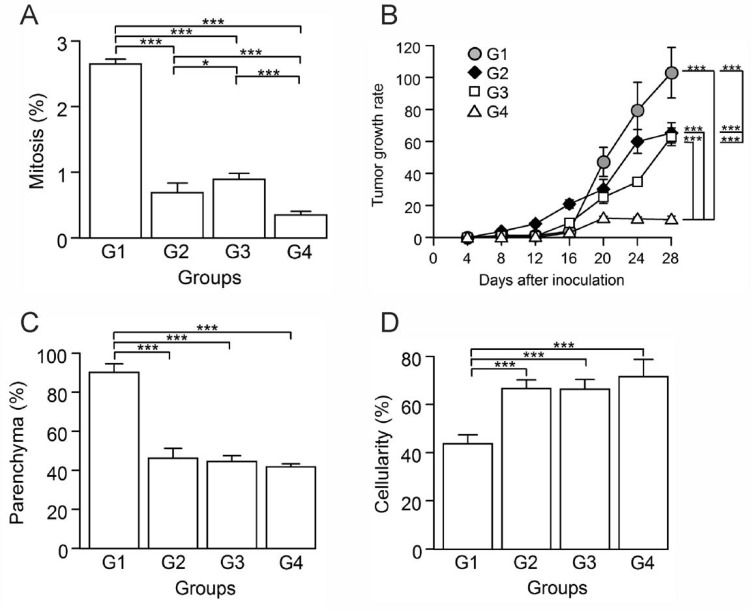
Autologous tumor cells/BCG/formalin vaccine inhibits tumor cell proliferation and the growth of 4T1 murine mammary tumors Tumor slides obtained from each mice group were stained with H&E to determine the percentage of mitosis, parenchyma and cellularity. (**A**) Significant decrease of mitosis percentage in G2, G3 and G4, compared to G1. (**B**) Tumor growth rate for each group presented as a percentage of volume increase with respect to the initial volume versus days after tumor induction. (**C**) Significant decrease of parenchyma percentage in G2, G3 and G4, compared to G1. (**D**) Significant increase of cellularity percentage in G2, G3 and G4 compared to G1. All data is shown as the mean percentage ± SEM of five mice per group. Tukey’s post hoc test results are shown (^*^p ≤ 0.05; ^**^p ≤ 0.01; ^***^p ≤ 0.001).

To assess the antitumor activity of the vaccine in our breast cancer model, we determined the mitosis percentage and cellularity through morphometric methods and utilized a calculated tumor growth rate as a measure of treatment effectiveness. An effective antitumor therapy should arrest tumor cell proliferation. In this sense, G4 showed the lowest mitosis percentage (Figure 2A), being 7.5 times lower than G1. G4 also showed the lowest tumor growth rate (Figure 2B), demonstrated by only an 11-fold net volume increase compared to a 102-fold increase in G1 (*p* ≤ 0.05). In all treated groups (G2, G3, G4), we observed that the tumor parenchyma diminished significantly (*p* ≤ 0.05) and about equally (Figure 2C), approximately 50% less than G1. Additionally, the cellularity increased significantly in G2, G3 and G4, with nearly twice more than in G1 (*p* ≤ 0.05) (Figure 2D). Despite similar necrosis and cellularity percentages, G2 and G3 had a tumor growth rate higher than G4. These results suggest that the vaccine administration induces a positive immune response, which leads to an important reduction of the tumor growth.

### Autologous tumor cells/BCG/formalin vaccine activates a strong antitumor immune response and establishes immunological memory

**Figure 3:**
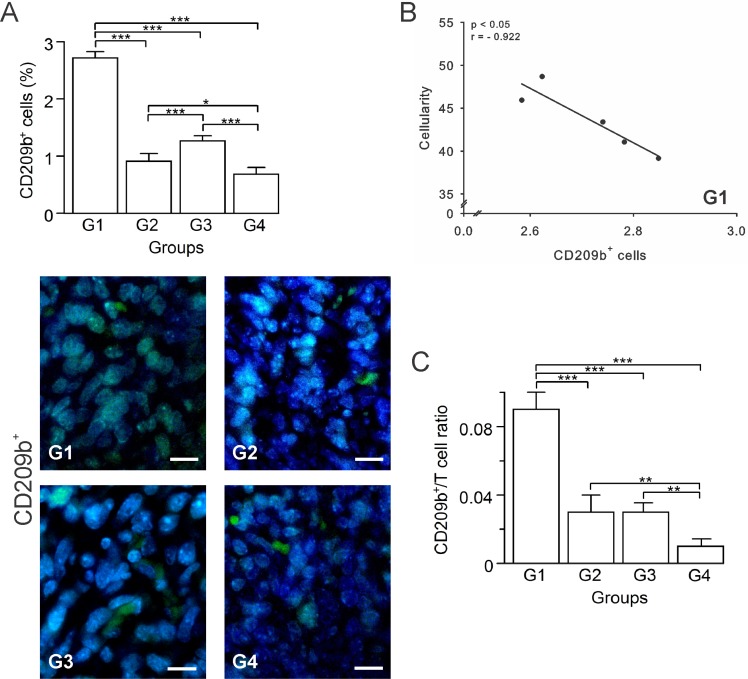
Autologous tumor cells/BCG/formalin vaccine changes the dynamics of tumor-infiltrating APCs in 4T1 breast cancer model Tumor slides obtained from each mice group were processed by immunohistochemistry to detect CD209b^+^ cells. (**A**) Significant decreased numbers of CD209b^+^ cells in G2, G3 and G4, compared to G1. Representative immunohistochemical staining of CD209b^+^ cells in all four groups studied are presented as CD209b^+^ cells in green and DAPI nuclear staining in blue. Scale bar, 10 μm. (**B**) Scatter diagram analysis showing a negative correlation between CD209b^+^ cells and percentage of cellularity in G1. (**C**) Dynamic changes of CD209b^+^ to CD8^+^ T cell ratios were analyzed based on the proportions of CD209b^+^ and CD8^+^ T cells. The data is shown as the mean ± SEM of five mice per group. Tukey’s post hoc test results are shown (^*^p ≤ 0.05; ^**^p ≤ 0.01; ^***^p ≤ 0.001).

Attempting to define the nature of the cell infiltrate in each group, we determined the percentage of several pro and antitumor cells in tumor sections. The activation of DCs is essential for the generation of an effective antitumor response, though when present in great quantities, they might exert protumor and anti-inflammatory effects [16]. In general, mature DCs have been considered immune-stimulatory, whereas immature DCs are suppressive and tolerogenic [17]. T-cell interactions with immature DCs can lead to T-cell tolerance through various mechanisms, including deletion, anergy, and the generation of regulatory T cells (Tregs) [18–21]. A protumor condition compatible with the presence of immature DCs prevailed in G1, which had the highest percentage of CD209b^+^ cells (Figure 3A). A negative correlation between these cells and cellularity (*p* ≤ 0.05, *r* = –0.922), suggests DCs’ immunosuppressive role in G1 (Figure 3B). In contrast, groups G2, G3 and G4, showed a significant decrease of CD209b^+^ cells compared to G1 (*p* ≤ 0.05) (Figure 3A). Additionally, to estimate T cells stimulation by antigen-presenting cells (APCs) we calculated the APC/T cell ratio using the quantification data of CD209b^+^ cells as APCs. DCs numbers and their state of maturation may affect their efficiency on priming T cells [17]. Therefore, this ratio is used as an indicator of CD8^+^ T and CD4^+^ T cells priming and proliferation by APCs. Low APC/T cell ratio suggests a greater expansion of effector T cells, while high APC/T cell ratio suggests an arrest of T cell expansion [22]. We found that G1 presented the highest ratio, followed by G2 and G3 both with the same value, and G4 with the lowest APC/T cell ratio (Figure 3C).

**Figure 4:**
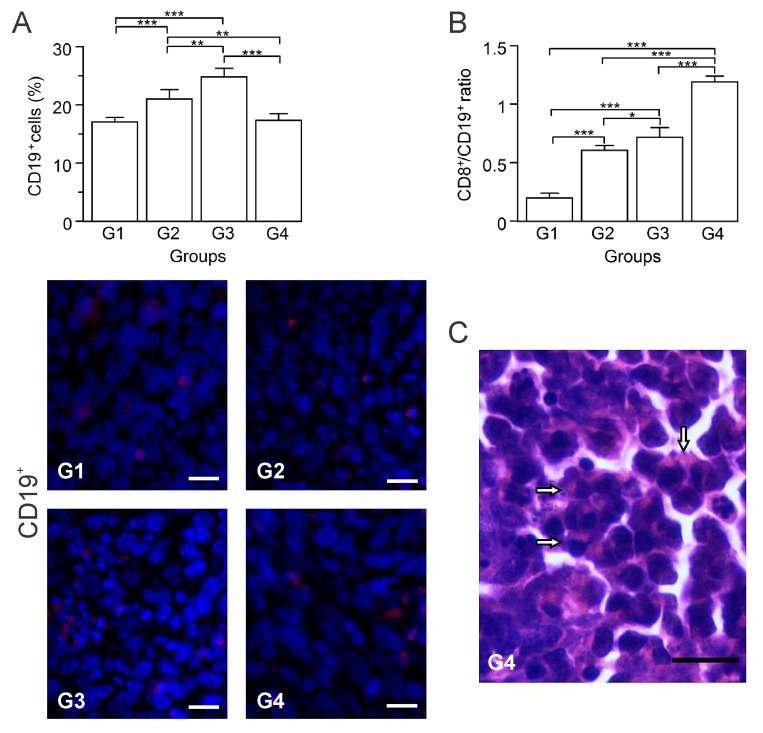
Autologous tumor cells/BCG/formalin vaccine shifts B cells population towards prevalence of effector B cells over Bregs Tumor slides obtained from each mice group were processed by immunohistochemistry to detect CD19^+^ cells. (**A**) Significant increment of CD19^+^ cells in G2 and G3, compared to G1 and G4. Representative immunohistochemical staining of CD19^+^ cells in all four groups studied are presented as CD19^+^ cells in red and DAPI nuclear staining in blue. Scale bar, 10 μm. (**B**) Dynamic changes of CD19^+^ to CD8^+^ T cell ratios were analyzed based on the proportions of CD19^+^ and CD8^+^ T cells. (**C**) Representative image of a H&E stained slide showing plasma cells infiltration into the tumor tissue of G4 (arrows). Scale bar, 15 μm. The data is shown as the mean percentage ± SEM of five mice per group. Tukey’s post hoc test results are shown (^*^ *p* ≤ 0.05; ^**^ *p* ≤ 0.01; ^***^p ≤ 0.001).

In response to an effective immunotherapy, B cells can be differentiated into plasma cells that produce antibodies against the tumor, inducing tumor cell-specific lysis and apoptosis [23, 24]. However, a particular subset of B cells, named regulatory B cells (Bregs), has been implicated in tumor growth and metastasis in different murine models of breast cancer [25–27]. In order to estimate B cells participation in the immune response, we quantified the CD19^+^ B cells for each group by immunohistochemistry. B cell numbers progressively increased in G2 and G3 relative to G1, whereas in G4, the numbers were similar to those seen in G1 (*p* ≥ 0.05) (Figure 4A). Given this result and to infer the nature of these B cells, we calculated the CD8^+^ T cells/B cells ratio (CTL/B ratio). High CTL/B ratios may be related to CTL-mediated tumor cells killing, thus limiting the tumor growth [28]. G1 showed the lowest CTL/B ratio, with progressively rising ratios for G2, G3, and G4 (Figure 4B). G4 showed a ratio 10-fold higher than G1 (*p* ≤ 0.05), suggestive of CTLs activation. In G2, correlations between CD19^+^ and CD8^+^ T cells (*p* ≤ 0.05, *r* = 0.950) suggest T cell priming by B cells (Supplementary Figure 1A). Presence of plasma cells in G3 and G4 tumor sections stained with H&E suggest the existence of antibody-secretor cells, though being more abundant in G4 (Figure 4C).

**Figure 5:**
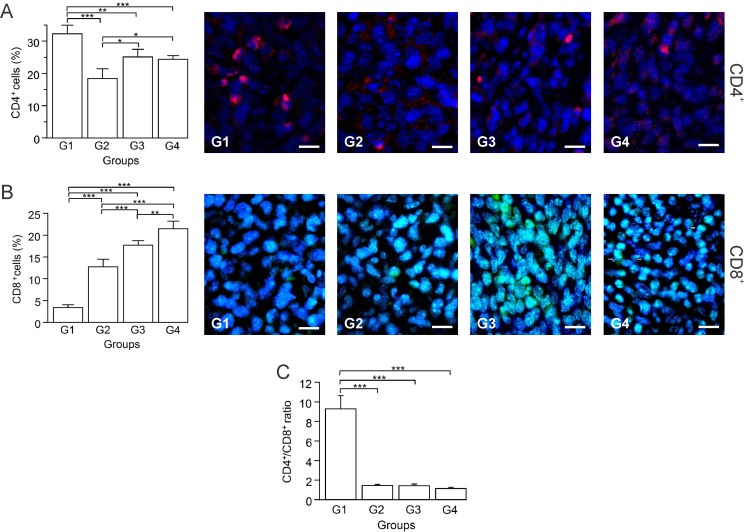
Autologous tumor cells/BCG/formalin vaccine induces an infiltration of CD8^+^ T cells and promotes prevalence of CD4^+^ Th1 cells over Tregs Tumor slides obtained from each mice group were processed by immunohistochemistry to determine CD4^+^ and CD8^+^ T cells. (**A**) Significant decreased numbers of CD4^+^ T cells in G2, G3 and G4, compared to G1. Representative immunohistochemical staining of CD4^+^ T cells in all four groups studied are presented as CD4^+^ T cells in red and DAPI nuclear staining in blue. Scale bar, 10 μm. (**B**) Significant increment of CD8^+^ T cells in G2, G3 and G4, compared to G1. Representative immunohistochemical staining of CD8^+^ T cells in all four groups studied are presented as CD8^+^ T cells in green and DAPI nuclear staining in blue, observing presence of CD8^+^ lymphocytes located close to tumor cells in tissue slides obtained from G4 (arrows). Scale bar, 10 μm. (**C**) Dynamic changes of CD4^+^ to CD8^+^ T cell ratios were analyzed based on the proportions of CD4^+^ and CD8^+^ T cells. The data is shown as the mean percentage ± SEM of five mice per group. Tukey’s post hoc test results are shown (^*^P ≤ 0.05; ^**^P ≤ 0.01; ^***^P ≤ 0.001).

CD4^+^ T and CD8^+^ T cells play essential roles in antitumor response. CD4^+^ T cells are critical for priming, inducing subsequent expansion and generation of memory of tumor-specific CD8^+^ T cells [29]. CD8^+^ T cells are key effector cells and have positive effects on antitumor immunity and patient clinical outcomes [30]. CD4^+^ T cells are comprised of two main populations: antitumor Th1 and anti-inflammatory regulatory T cells (Tregs) [30]. A heavy Treg cell accumulation within breast tumor tissues has been reported in various studies, identifying it as a possible mechanism used by tumor cells to evade host immune responses from CD8^+^ T cells [31–33]. Based on the importance of the balance of T cells in the antitumor response, we determined by immunohistochemistry the percentage of CD4^+^ and CD8^+^ T cells. G1 had the highest CD4^+^ T cell count, followed by consecutively lower counts in G3, G4 and G2 (Figure 5A). In contrast, G4 showed a marked increase of CD8^+^ T cells with G3, G2 and G1 having progressively less cells (Figure 5B). In G4, CD8^+^ T cells were close to the tumor cells, primarily in Z1 (Figure 5B). High CD4/CD8 ratios in breast cancer patients were strongly associated with worse prognosis [30, 34]. Thus, we calculated the CD4/CD8 ratio, keeping in mind that for positive results this ratio should be kept as low as possible. G1 presented the highest ratio in contrast with the treated groups (G2, G3, G4), which showed a marked reduction (Figure 5C). In G2, CD4^+^ T cells were positively correlated with the mitosis percentage (*p* ≤ 0.05, *r* = 0.884), and tumor growth rate (*p* ≤ 0.05, *r* = 0.966), suggesting prevalence of Tregs (Supplementary Figure 1B–1C). A low number of probable remaining Tregs in G3, could be counteracted by IFN-γ, as indicated by a negative correlation between CD4^+^ T and IFN-γ^+^ cells (*p* ≤ 0.05, *r* = –0.973) (Supplementary Figure 1D).

**Figure 6:**
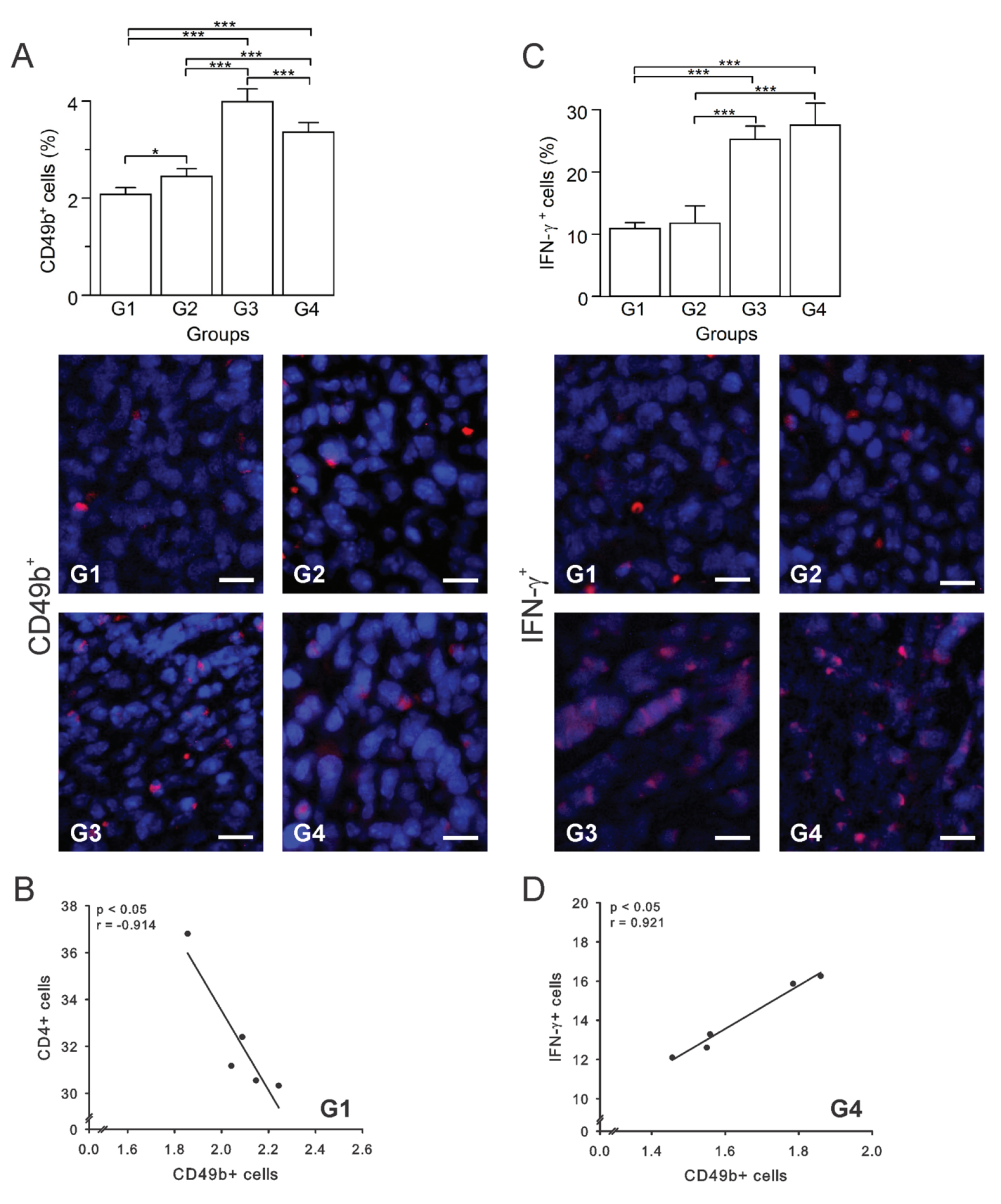
Autologous tumor cells/BCG/formalin vaccine induces an infiltration of NK cells and IFN-γ^+^ cells Tumor slides obtained from each mice group were processed by immunohistochemistry to determine CD49b^+^ and IFN-γ^+^ cells. (**A**) Significant increment of CD49b^+^ cells in G2, G3 and G4, compared to G1. Representative immunohistochemical staining of CD49b^+^ cells in all four groups studied are presented as CD49b^+^ cells in red and DAPI nuclear staining in blue. Scale bar, 10 μm. (**B**) Scatter diagram analysis showing a negative correlation between CD49b^+^ and CD4^+^ T cells in G1. (**C**) Significant increment of IFN-γ^+^ cells in G3 and G4, compared to G1 and G2. Representative immunohistochemical staining of IFN-γ ^+^ cells in all four groups studied are presented as IFN-γ^+^ cells in red and DAPI nuclear staining in blue. Scale bar, 10 μm. (**D**) Scatter diagram analysis showing a positive correlation between CD49b^+^ cells and IFN-γ^+^ cells in G4. The data is shown as the mean percentage ± SEM of five mice per group. Tukey’s post hoc test results are shown (^*^P ≤ 0.05; ^**^P ≤ 0.01; ^***^P ≤ 0.001).

NK cells are lymphocytes present in the innate immune system and constitute the first line of defense against viruses and tumors [35]. In cancer, once the tumor cell is recognized, NK cells trigger their own cytotoxic activity or release cytokines and recruit other immune cells by IFN-γ and tumor necrosis factor-alpha (TNF-α) secretion [35]. It is known that BCG immunotherapies enhance NK cells recruitment [36]. However, as it has been demonstrated in different tumor types, the NK cell antitumor function can be negatively affected by the presence of Tregs [37–39]. In this study, G1 and G3 presented the lowest and highest percentages of NK cells (CD49b^+^), respectively; whereas G2 and G4 showed intermediate values (Figure 6A). Given this somewhat unexpected result, we calculated several correlations to attempt to clarify it. We found that in G1 there was a negative correlation between CD49b^+^ and CD4^+^ T cells (*p* ≤ 0.05, *r* = –0.914) (Figure 6B), indicating that CD4^+^ T cells, which were likely Tregs, might be inhibiting the proliferation/function of NK cells. Additionally, the IFN-γ^+^ cells are known to be a significant factor in tumor elimination. Here we determined that G1 and G2 had the lowest IFN-γ^+^ cells percentage (*p* ≥ 0.05) (Figure 6C), while G3 and G4 showed the highest values (*p* ≥ 0.05) (Figure 6C). Furthermore, in G4 CD49b^+^ cells were positively correlated with IFN-γ^+^ cells (*p* ≤ 0.05, *r* = 0.921) (Figure 6D). Altogether, these data suggest that the high infiltration of cells into tumors of G4 animals is mainly composed of immune cells with antitumor properties (CD4^+^ Th1, CD8^+^ T and NK cells), and that the vaccine might also be inducing immune memory.

### Tumor-associated macrophages (TAMs) and myeloid-derived suppressor cells (MDSCs) recruitment is reduced by autologous tumor cells/BCG/formalin vaccine

**Figure 7:**
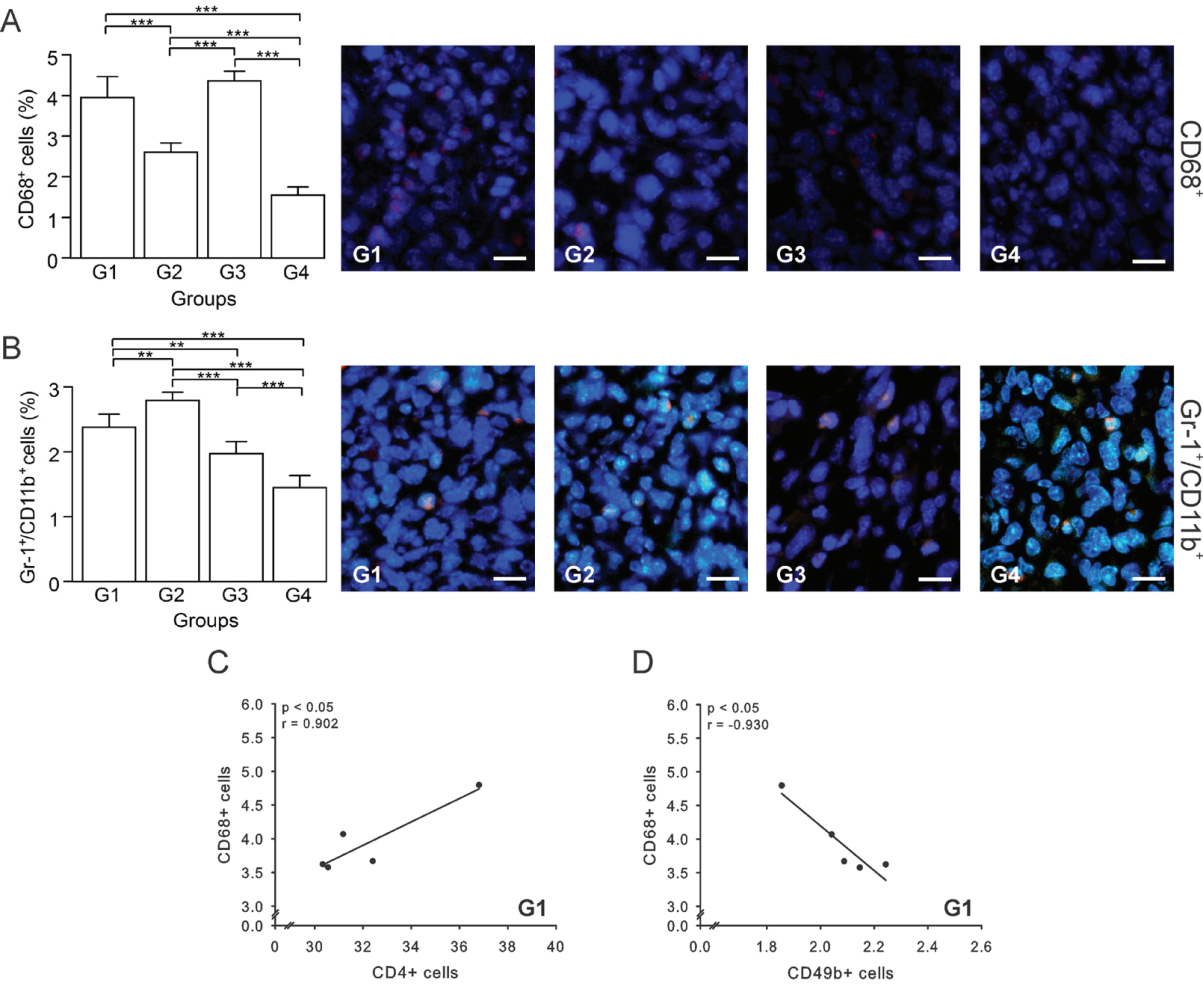
Autologous tumor cells/BCG/formalin vaccine reduces TAMs and MDSCs recruitment Tumor slides obtained from each mice group were processed by immunohistochemistry to determine CD68^+^ (TAMs) and Gr-1^+^/CD11b^+^ cells (MDSCs). (**A**) Significant decreased numbers of CD68^+^ cells in G2 and G4, compared to G1. Representative immunohistochemical staining of CD68^+^ cells in all four groups studied are presented as CD68^+^ cells in red and DAPI nuclear staining in blue. Scale bar, 10 μm. (**B**) Significant decreased numbers of Gr-1^+^/CD11b^+^ cells in G3 and G4, compared to G1. Representative immunohistochemical staining of Gr-1^+^/CD11b^+^ cells in all four groups studied are presented as Gr-1^+^/CD11b^+^ cells in yellow and DAPI nuclear staining in blue. Scale bar, 10 μm. (**C**) Scatter diagram analysis showing a positive correlation between CD68^+^ and CD4^+^ T cells in G1. (**D**) Scatter diagram analysis showing a negative correlation between CD68^+^ cells and CD49b^+^ cells in G1. The data is shown as the mean percentage ± SEM of five mice per group. Tukey’s post hoc test results are shown (^*^p ≤ 0.05; ^**^p ≤ 0.01; ^***^p ≤ 0.001). Scale bar, 10 μm.

An immunotherapy is usually considered successful when it prevents infiltration of protumor cells, such as TAMs and MDSCs [40]. Thus, we determined the percentage of TAMs (CD68^+^ cells) and MDSCs (Gr-1^+^/CD11b^+^ cells) in tumor sections by immunohistochemistry. Our results showed that G3 had the highest percentage of TAMs, while G4 showed the lowest values, with G1 and G2 being intermediate (Figure 7A). In the case of MDSCs, G2 had the highest percentage with G1, G3, and G4 showing progressively lower values; the latter showing almost a 2-fold reduction with respect to G1 (Figure 7B). For both, TAMs and MDSCs, G4 showed the lowest values, suggesting that when using this index of immunotherapeutic effectiveness, our vaccine could be considered successful. Additionally, in G1 TAMs were positively correlated with CD4^+^ T cells (*p* ≤ 0.05, *r* = 0.902) (Figure 7C), but negatively correlated with NK cells (*p* ≤ 0.05, *r* = –0.930) (Figure 7D), suggestive of an immunosuppressive role of TAMs. Furthermore, a correlation between TAMs and CD8^+^ T cells (*p* ≤ 0.05, *r* = –0.982) (Supplementary Figure 1E) support a protumor function of TAM cells in G3. In sum, these results suggest that the vaccine affects the recruitment of TAMs and MDSCs, thus diminishing their numbers into the tumor.

## DISCUSSION

The autologous tumor cells/BCG/formalin vaccine was previously described in a non-randomized observational experience, which suggested promise for its use in immunotherapy for breast cancer [13–15]. Here, we used a murine 4T1 breast cancer model to demonstrate that the vaccine is indeed effective and to obtain mechanistic data. The high promise of our descriptive human experience was supported by our animal model in that the vaccination group (G4) had a significant reduction in tumor growth and the possible establishment of an immune memory response, as indicated by the presence of plasma cells in the tumors. Additionally, we acquired important information about the cellularity in the tumor microenvironment, which points to some of the processes that occur in the treated animals.

Mice in the G4 group had correspondingly 10 and 5 times lower tumor growth rates than those observed in the vehicle control group (G1) and other treated groups (G2 and G3). The lowest mitotic index was also present in G4, showing effective control of tumor growth. These differences in tumor growth rate and mitotic index were likely determined by the varying types of cells that infiltrated the tumors in each group. The infiltrating cell types might depend on the status of the BCG and tumor antigens, which is likely influenced by the presence or absence of the diluted formalin in the formulation. In G2 and G4, the BCG was inactivated by the formalin solution, in contrast to G3, where the mycobacteria were alive. This was demonstrated by the BCG being located extracellularly in G2 and G4, whereas in G3, the BCG was located inside phagocytes. Similarly, in G3 tumor antigens were in their native state, whereas in G2 and G4 they were chemically modified by the formalin.

G1 presented a pattern of coagulative necrosis likely caused by tissue hypoxia due to accelerated tumor growth, insufficient neovascularization, compression and thrombotic obstruction of adjacent vessels [41]. In the treated groups, necrosis was not due to excessive growth and vascular insufficiency, but likely due to activation of immunological cells with either the live BCG (G3) or formalin-attenuated BCG (G2 and G4). The activation via TLR2, TLR4 and TLR9 enhances IFN-γ levels [6, 42], driving chemotaxis of neutrophils, DCs, and macrophages to the inoculation site, where they internalize the bacilli and/or tumor antigens, and spread through the lymphatic vessels [43]. Neutrophils infiltrating the tumor, by means of TRAIL expression, induce apoptosis and necrosis of tumor cells [42]. As a result of necrosis, damage-associated molecular patterns (DAMPs) are released and interact with pattern recognition receptors (PRRs), inducing the expression of cytokines that stimulate DCs. Subsequently, these cells efficiently take up and process tumor antigens, giving rise to cross-prime T cells [44–46]. This type of immune response occurred more robustly in G4, probably due to the presence of tumor antigens that were modified by the diluted formalin in the vaccine. In G2, the initial necrosis induced by the BCG, might have induced the release of tumor antigens, but not sufficiently to trigger the type of strong immunological response via APCs and T cells observed in G4, where both BCG and tumor cells homogenate where modified by the diluted formalin.

IFN-γ is known to be the main pro-inflammatory cytokine involved in antitumor T cell activation and direct tumoricidal activity [47]. This cytokine is produced at high levels in response to live BCG [48]. Although in G4 BCG is inactivated, it still produces a marked increase of IFN-γ^+^ cells, which seems to be aligned with the high number of CD8^+^ T and NK cells present in this group’s tumor samples. A protective antitumor immunity depends on DCs’ antigen presentation to CD8^+^ T and B cells via MHC I molecules [49]. Additionally, CD4^+^ T cells participation is important in tumor control, as CD4^+^ Tregs are associated with tumor progression, whereas CD4^+^ Th1 with an antitumor immune response [50]. The activation of CD8^+^ T and B cells against the tumor is conditioned by the APCs/T cells proportion and DCs state of maturation. At different stages of tumor growth, differences in the type, phenotype and amount of tumor infiltrating DCs (TIDCs) are present [17]. Animal models of ovarian cancer have shown that as cancer progresses, the number of TIDCs increases, but switches from immune stimulatory to immune suppressive DCs [51, 52]. This pattern was associated to an important loss of T cell infiltration [52]. Similarly, patients with disease recurrence of colorectal cancer present higher densities of immature TIDCs and lower densities of mature TIDCs in their primary lesions [53]. One of the possible mechanisms by which immature DCs block adaptive immune responses is the induction of Tregs [54]. DCs, specifically in breast cancer, have shown to enhance Treg expansion, contributing to immune tolerance and adverse clinical outcomes [55]. Additionally, in pancreatic and breast cancer, Treg cell-mediated DCs suppression could be induced by direct cell-to-cell contact, leading to apoptosis and downregulation of costimulatory molecules in DCs [56, 57]. In our study, G4 showed a 9-fold reduction in the APC/T cells ratio, in contrast to the high ratio and immunosuppression-associated correlation observed in G1, as well as a 3-fold lower APC/T cell ratio compared to G2 and G3. This result allows us to infer that in G4, APCs might have effectively primed CD8^+^ T cells; in contrast with G1, G2, and G3, which reveal a presumptive impaired ability of APCs to present antigens to T cells. Furthermore, the reduction in DCs in G4 was associated to a high CD8^+^ T cells percentage resulting from a probable CD4^+^ Treg depletion and CD4^+^ Th1 predominance. Regarding these results and all the findings in G1, Huang *et al.* (2015) [30] reported in a murine 4T1 tumor model at the same late stage of development evaluated in our study, a high infiltration of CD4^+^ T cells, mainly composed of Tregs, as well as high CD4/CD8 and Treg/CD8^+^ T cell ratios. Additionally, in breast cancer patients, these cell infiltration and ratios increments were positively correlated with advanced tumor stage, large tumor sizes and positive tumor metastasis, and inversely related with relapse-free and overall survival durations [30]. Similar results were also found in colorectal cancer, ovarian cancer, oropharyngeal squamous cell carcinoma and Hodgkin lymphoma [58–61]. Thus, the effectivity of our vaccine is supported by the diminishment of the CD4/CD8 ratio in G4 in comparison to G1, which suggests that the main subset of CD4^+^ T cells in G4 might be Th1 cells, and that this low ratio could be at the expense of Tregs diminution. In addition, the observation that in G4 CD8^+^ T cells are in close contact to tumor cells could reflect the main mechanism limiting tumor growth, as has been previously reported in medullary breast carcinoma patients [28]. Overall, our data supports that the tumor regression in G4 might be related to a Th1 immune response activation. Nevertheless, more specific determinations to identify Th1 cells and Tregs should be performed in future related studies.

NK cells are an essential innate immune cell subset and a main IFN-γ source. In BCG immunotherapy for bladder cancer, NK cells are the main effector in tumor elimination [62]. *In vitro* and *in vivo* studies using *M. tuberculosis* and BCG have demonstrated Treg lysis by NK cells [63, 64]. Therefore, the reduction in CD4^+^ T cell percentage observed in G4 could be associated with a marked NK cell increment that interferes with Tregs through IFN-γ secretion [64]. In this group (G4), the IFN-γ production might be mainly due to the high number of NK cells, evidenced by a positive correlation between CD49b^+^ and IFN-γ^+^ cells in our data. This IFN-γ secretion promotes an increase in CD8^+^ T cell recruitment, which together with NK cells, are directly implicated in tumor elimination.

B cells also play a fundamental role in anti-cancer immunity, especially plasma cells, which along with T cells, are part of the tumor-infiltrating lymphocytes (TIL) [65]. B cells induce breast cancer regression by secreting immunoglobulin G in response to tumor cells, and activating T cells to produce cytotoxic effects and IFN-γ [24]. Nonetheless, a subset of B cells, known as Bregs, have the opposite effect and promote carcinoma development. This is likely mediated by Treg induction, which is promoted by B cell-associated transforming growth factor beta (TGF-β) secretion [27, 66]. High CTL/B ratio in atypical medullary and medullary breast carcinoma was associated with a good prognosis as indicated by the absence of nodal metastases [28]. In our study, G1 showed the lowest CTL/B ratio suggestive of Breg predominance and a high number of CD4^+^ T cells, which were related to the tumor expansion observed. In contrast, the activation of a Th1 immune response in G4 is compatible with the presence of effector B cells and tumor growth reduction demonstrated by the highest CTL/B ratio. While in future studies more specific assays for B cells identification should be performed, our results, and the presence of plasma cells infiltrating the tumor in G4, are suggestive of a generation of immunological memory [65, 67].

For an effective antitumor response, infiltration of pro-inflammatory and cytotoxic cells is necessary, as well as low levels of protumor cells, such as TAMs and MDSCs [40]. Two different populations of TAMs have been described: M1 macrophages that are pro-inflammatory and recruit Th1 cells; and M2 macrophages that inhibit antitumor T lymphocytes, express anti-inflammatory molecules, and recruit Tregs, all contributing to tumor tolerance [40, 68]. IFN-γ has the ability to switch immunosuppressive TAMs into immunostimulatory cells, block the production of protumor factors by TAMs, and prevent TAMs generation [68]. G4 had significantly lower percentage of TAMs with respect to all other groups. A high number of IFN-γ^+^ cells in G4 might have promoted the conversion of M2 TAMs into M1 TAMs, whereas G1 had an opposite pattern. In G1, a possible predominance of M2 TAMs could be responsible of CD4^+^ T cells differentiating toward Tregs, as it was suggested by a positive correlation between these two cell types in this group. However, further specific identification of M1 and M2 TAMs is necessary to directly show this as a mechanism. MDSCs are immature myeloid Gr1^+^/CD11b^+^ cells that inhibit the cytotoxic functions of NK, B, T and DCs [40]. MDSCs cells promote tumor growth by several mechanisms including their inherent immunosuppressive activity, promotion of neo-angiogenesis, mediation of epithelial-mesenchymal transition and alteration of cancer cell metabolism [40]. They also promote Treg proliferation and inhibit DCs maturation by secreting immunosuppressive cytokines [40, 69]. In G4, tumor antigens were part of the treatment, leading to an antigen-based response. This might have inhibited the recruitment of MDSCs, where the immune response was amplified as indicated by the low CD4/CD8 and APC/T cell ratios, suggesting the development of an effective antitumor Th1 response.

An important factor in the effectiveness of the vaccine was likely the modification of BCG and tumor antigens immunogenicity by low concentrations of formaldehyde. This idea, proposed by Convit & Ulrich in 2006 [13], prevents the deleterious effects of live BCG on the APC response [70], amplifying it via TLR. Depending on its concentration, formalin is known to have adverse effects in vaccines inactivated by this compound, or induce reactive carbonyl groups on vaccine antigens, which has shown to enhance immunogenicity and efficacy without necessarily causing adverse effects [71]. Low concentrations, although denaturing proteins, conserve oligosaccharide epitopes, which are important in the immunological response [72]. The conservation of these epitopes enhances the specificity of the immunological response toward oligosaccharides, which are recognized by APCs, increasing antigen uptake and activating several intracellular signaling pathways. This results in cytokine secretion, cell activation, phagocytosis and antigen presentation that leads to the differentiation of CD4^+^ T cells and the activation of adaptive immune responses [73].

In contrast with the antitumor effectiveness observed in G4, our findings in the G2 and G3 groups indicate that all three components of the vaccine are necessary for an optimal tumor growth reduction. In G2, although the BCG was attenuated, the absence in the injected preparation of tumor specific antigens might have been responsible for defective APCs activation, high level of MDSCs, and probably Tregs predominance and Bregs infiltration. This demonstrates that BCG plus formalin alone are insufficient to generate an effective immune antitumor response in this breast cancer model. In G3, given the absence of low dose formalin, the BCG was alive and tumor antigens were in their native form. Although G3 generated high levels of IFN-γ^+^ and NK cells, as well as probable CD4^+^ Th1 cells predominance, the antitumor immune response was not as potent as in G4, likely due to a negative effect of live BCG on APCs [70, 74]. Also, native tumor antigens result in a lower specificity of immunological response. Therefore, APCs with impaired functionality as well as the presence of tumor antigens in native form may have caused TAMs M2 and Breg recruitment plus a poor CD8^+^ T cell activation in G3, thus emphasizing diluted formalin as a key component of the more effective treatment.

Taken together, our results indicate that the autologous tumor cells/BCG/formalin vaccine induces a strong immune activation characterized by an important infiltration of cytotoxic (CD8^+^ T and NK cells) and B cells. This process, in conjunction with a reduced infiltration of immunosuppressive cells, and a probable predominance of CD4^+^ Th1 cells, effectively reduces tumor growth and promotes the establishment of immune memory. This response was evidenced by a low tumor growth rate, diminution of tumor mitotic index, extensive necrosis and plasma cells infiltration. All these favorable immunological responses, in addition to its very low cost of production, ease of preparation, and an apparent favorable safety profile as seen in the previous clinical experience [15], support our vaccine approach as an excellent candidate for a highly accessible personalized immunotherapy for breast cancer.

We suggest that this immunotherapy be tested in combination with other treatments and be considered for further studies. Research on the safety and the induced immunity mechanism of our vaccine must continue, as well as its future consideration for a human trial application.

## MATERIALS AND METHODS

### Mice and cell line

Since breast cancer prevalence is very low in males, we only utilized female mice. Six- to eight-week-old female BALB/c mice were provided by Escuela de Medicina José María Vargas (Universidad Central de Venezuela) and maintained in their animal facility. The 4T1 cell line was provided by the Cellular and Molecular Pathology Laboratory at IVIC and maintained in the recommended medium. This study was approved by the Bioethics Committee of Escuela de Medicina José María Vargas.

### Tumor induction and preparation of autologous tumor cells/BCG/formalin vaccine

Ten mice were inoculated with 4T1 cells to obtain tumor tissue to prepare autologous tumor cells homogenate. The 4T1 cells were harvested using 0.25% trypsin (Sigma-Aldrich) in 0.05% EDTA (EMD Millipore Corporation), washed once with RPMI-1640 (MP Biomedicals LLC) and re-suspended in 1× PBS (4.45 mM Na_2_HPO_4_, 1.55 mM NaH_2_PO_4_, 137 mM NaCl, pH 7.2). Viability of cells was determined by 0.25% trypan blue dye exclusion. 4T1 cells (1 × 10^6^ cell/mouse) were injected subcutaneously (s.c.) into the mammary fat pad of mice. When tumors reached a volume of 1.5–2.5 cm^3^, mice were anesthetized with xylazine/ketamine until unresponsive to toe tap and/or agonal breathing, and then euthanized to obtain the tumors. Primary tumors were extracted in sterile conditions and stored in PBS plus penicillin-streptomycin 1× (Sigma-Aldrich) at –80° C until their use. On the same day of vaccination, the tumors were processed following the protocol proposed by Convit *et al.* (15) with minor modifications. Briefly, tumors (0.5 gr) were washed with PBS plus penicillin-streptomycin 1× and then mechanically macerated in a homogenizer with sterile PBS (1 mL). The tumor cells homogenate was centrifuged for 10 minutes at 250 × g and the supernatant collected. Proteins were quantified by Bradford´s method. The final vaccine mixture containing 200 µg tumor cells homogenate, 0.0625 mg BCG, and 0.02% formaldehyde in a final volume of 100 µL was prepared as previously described by Convit *et al.* [15] and administered immediately after its preparation.

### Tumor model used, study treatment groups, measurement of tumor volume, and calculation of tumor growth rate

#### Tumor model

A total of 1 × 10^6^ 4T1 cells were injected s.c. into the mammary fat pad of female BALB/c mice.

#### Study treatment procedures and treatment groups

20 BALB/c mice were randomly assigned to four groups, five animals each. The treatments were initiated five days post tumor induction and consisted of 100 µL of the corresponding treatment injected intradermally on the base of the neck once a week for four weeks. The four treatment groups were defined as follows: Group 1 (G1) control treated with PBS; Group 2 (G2) treated with BCG plus formalin (0.0625 mg BCG and 0.02% formaldehyde); Group 3 (G3) treated with autologous tumor cells homogenate plus BCG (200 µg tumor cells homogenate and 0.0625 mg BCG); and Group 4 (G4), our vaccination group, treated with the autologous tumor cells/BCG/formalin (200 μg tumor cells homogenate, 0.0625 mg BCG, and 0.02% formaldehyde) (Table 1).

#### Measurement of tumor volume

Tumor volumes were measured every 4 days up to 28 days post tumor induction, calculated as shown by Feldman *et al.*, (2009) [75] and expressed in mm^3^.

#### Calculation of the tumor growth rate

The tumor growth rate is defined as the percentage of volume increase relative to the initial volume.

### Histology and immunohistochemistry

Five weeks post-tumor induction, all mice were euthanized and tumors extracted and subjected to a pathologic examination. Tissues were fixed in 4% w/v formaldehyde, paraffin-embedded. Sections at 5 µm interval were cut and stained with Hematoxylin and Eosine (H&E), and Gomori´s trichome. The immunohistochemistry was carried out according to Mihara *et al.* (2011) [76]. The following primary antibodies were used: goat polyclonal anti-mouse CD209b (Santa Cruz Biotechnology, Cat No. sc-25221), rat monoclonal anti-mouse CD49b (Biolegend, Cat No. 108901), rat monoclonal anti-mouse CD68 (Biolegend, Cat No. 137001), rat monoclonal anti-mouse CD4 (Biolegend, Cat No. 100505), rat monoclonal anti-mouse CD19 (Biolegend, Cat No. 115501), rat monoclonal anti-mouse CD8-α (Santa Cruz Biotechnology, Cat No. sc-18913), rat monoclonal anti-mouse Ly-6G/Ly-6C (Gr-1) (Biolegend, Cat No. 108401), rabbit polyclonal anti- Integrin αM (CD11b) (Santa Cruz Biotechnology, Cat No. sc-28664) and rat anti-mouse IFN-γ (Biolegend, Cat No.505701). The secondary antibodies used were the following: goat polyclonal anti-rabbit IgG coupled with fluorescein isothiocyanate (FITC) (Abcam Inc., Cat No. ab6717), goat polyclonal anti-mouse IgG labeled with tetramethyl-rhodamine isothiocyanate (TRITC) (Abcam Inc., Cat No. ab6897), sheep anti-rat IgG labeled with TRITC (Abcam Inc., Cat No. ab6849), goat polyclonal anti-rabbit IgG coupled with FITC (Abcam Inc., Cat No. ab6717), mouse polyclonal anti-goat IgG coupled with FITC (Santa Cruz Biotechnnology, Cat No. sc-2356), and goat polyclonal anti-rabbit IgG coupled with Texas red (Santa Cruz Biotechnology, Cat No. sc-2780). The nuclei were stained with DAPI and the slides mounted with a DABCO containing medium.

Observation and imaging of immunolocalization was performed according to Iwamoto & Allen (2004) [77] with slight modifications as follows. The observation was carried out using a fluorescent and light microscope Eclipse E600 (Nikon) equipped with epifluorescence illumination. Slides were digitally photographed with a SPOT Flex FX1520 camera (SPOT Imaging). Images of 2048 × 2048 pixels were saved as 24-bit color TIFF files. The ImageJ software (version 1.46r) (National Institute of Health, Bethesda, MA, USA) was used for image analysis. Immunofluorescence positive cell counting was carried out in 6 aleatory areas in one section per specimen per mouse. The total count of positive cells for each antigen was calculated taking into consideration the average cellularity value obtained in each mice group. Background fluorescence was eliminated using the subtract background function of the software. For this, control sections were treated only with PBS, using the same illumination conditions and digital camera settings; then the respective background level was subtracted.

### Determination of cellularity

Cellularity was determined by image analysis in 5 aleatory areas (40×) on H&E-stained sections, in three different sections for each specimen per mouse (*n* = 15). All cell nuclei in the RGB layer were counted using the ImageJ software. Briefly, images were converted to an 8-bit format and then adjusted for contrast, brightness, and threshold. The cells in each field were counted individually by enclosing the region of interest with the selection tool and using the option Analyze Particles of ImageJ. Results were expressed as number of cells/1000 μm^2^ [78].

### Determination of mitotic index

The H&E-stained sections were examined at total magnification 1000×, using immersion oil to enable the recognition of mitotic figures with high accuracy. Only metaphases, anaphases, and telophases were counted, as well as the number of nuclei. The mitotic index is then defined as the number of mitoses per one hundred cells expressed as percentage and calculated as follows:

Mitotic index=(Number of mitoses per unit area/Number of nuclei per unit area)×100

The counted areas were selected randomly, ensuring that only stroma was present in the observed field [79].

### Quantification of necrosis and parenchyma in tumors

The necrotic areas were determined in tumor sections stained with H&E following the protocol proposed by Moffitt (1994) [80]. Images were converted from 24 to 8 bits, brightness and contrast were increased to differentially observe the tumor parenchyma from the necrotic zones and the color range threshold was set to exclusively encompass this differentiation. The images were finally converted to one byte black and white images. Values above the target threshold resulted in white pixels, whereas values that fell on or below the target threshold resulted in groups of black pixels, representing the necrotic areas. The ‘Analyze Particles’ option of ImageJ was set to accept between 1 and 10,000 pixels, in order to include features that are not separable from each other, but excluding large non-necrotic areas. Under these conditions, ImageJ provided the number, total area, and fractional area (percent) occupied with necrotic zones in the pictures. The subtraction of the area occupied by necrotic cells from the total area constituted the parenchyma percentage.

### BCG identification

The identification of BCG was carried out by the method of conventional staining Ziehl-Neelsen and fluorescense, using acridine orange and auramine O for paraffin sections after Mote *et al.* (1975) [81].

### Statistical analysis

Kruskal-Wallis non-parametric tests were performed followed by Tukey’s post hoc tests. Pearson’s correlation tests were used to ascertain the associations. The PAST statistical program was used, and statistical significance was met by an α level of 0.05, two-tailed.

## SUPPLEMENTARY MATERIALS FIGURE



## Abbreviations

APCs: antigen-presenting cells
APC/T: antigen-presenting cells/ T cell ratio
BCG: bacillus Calmette-Guérin
Bregs: regulatory B cells
CTL: cytotoxic T lymphocyte
CTL/B: cytotoxic T lymphocyte/ B cell ratio
DABCO: 1,4-diazabicyclo[2.2.2]octane
DAMPs: damage associated molecular patterns
DCs: dendritic cells
DAPI: 4′,6-diamidino-2-phenylindole
EDTA: Ethylenediaminetetraacetic acid
FDA: Food and Drug Administration
FITC: fluorescein isothiocyanate
H&E: Hematoxylin and Eosine
MDSCs: myeloid-derived suppressor cells
MHC: major histocompatibility complex
NK: Natural Killer
PBS: phosphate buffer saline
PRRs: pattern recognition receptors
s.c.: subcutaneously
TAMs: Tumor-associated macrophages
TGF-β: transforming growth factor beta
TIL: tumor-infiltrating lymphocytes
TLRs: toll-like receptors
TRAIL: TNF-related apoptosis-inducing ligand
Tregs: regulatory T cells
TRITC: tetramethyl-rhodamine isothiocyanate
Z1: zone 1
Z2: zone 2

## References

[1] Ferlay J, Soerjomataram I, Ervik M, Dikshit R, Eser S, Mathers C, Rebelo M, Parkin DM, Forman D, Bray F http://globocan.iarc.fr.

[2] Kurtz SL, Ravindranathan S, Zaharoff DA (2014). Current status of autologous breast tumor cell-based vaccines. Expert Rev Vaccines.

[3] Temizoz B, Kuroda E, Ishii KJ (2016). Vaccine adjuvants as potential cancer immunotherapeutics. Int Immunol.

[4] Morales A, Eidinger D, Bruce AW (1976). Intracavitary Bacillus Calmette-Guerin in the treatment of superficial bladder tumors. J Urol.

[5] Lamm DL, Blumenstein BA, Crawford ED, Montie JE, Scardino P, Grossman HB, Stanisic TH, Smith JA, Sullivan J, Sarosdy MF, Crissman JD, Coltman CA (1991). A randomized trial of intravesical doxorubicin and immunotherapy with bacille Calmette-Guérin for transitional-cell carcinoma of the bladder. N Engl J Med.

[6] Redelman-Sidi G, Glickman MS, Bochner BH (2014). The mechanism of action of BCG therapy for bladder cancer—a current perspective. Nat Rev Urol.

[7] Berd D, Mastrangelo MJ (1988). Effect of low dose cyclophosphamide on the immune system of cancer patients: depletion of CD4+, 2H4+ suppressor-inducer T-cells. Cancer Res.

[8] Vermorken JB, Claessen AM, van Tinteren H, Gall HE, Ezinga R, Meijer S, Scheper RJ, Meijer CJ, Bloemena E, Ransom JH, Hanna MG, Pinedo HM (1999). Active specific immunotherapy for stage II and stage III human colon cancer: a randomised trial. Lancet.

[9] Soliman H (2010). Developing an effective breast cancer vaccine. Cancer Control.

[10] Convit J, Pinardi ME, Rodríguez Ochoa G, Ulrich M, Avila JL, Goihman M (1974). Elimination of Mycobacterium leprae subsequent to local *in vivo* activation of macrophages in lepromatous leprosy by other mycobacteria. Clin Exp Immunol.

[11] Convit J, Aranzazu N, Pinardi ME, Ulrich M (1979). Immunological changes observed in indeterminate and lepromatous leprosy patients and Mitsuda-negative contacts after the inoculation of a mixture of Mycobacterium leprae and BCG. Clin Exp Immunol.

[12] Convit J, Ulrich M, Aranzazu N, Castellanos PL, Pinardi ME, Reyes O (1986). The development of a vaccination model using two microorganisms and its application in leprosy and leishmaniasis. Lepr Rev.

[13] Convit J, Ulrich M (2006). [Desarrollo de una autovacuna + BCG y su posible uso en el tratamiento del cáncer]. [Article in Spanish]. Gac Med Caracas.

[14] Convit J (2008). [Inmunidad celular y su importancia en el cáncer de la mama]. [Article in Spanish]. Gac Med Caracas.

[15] Convit J, Montesinos H, Oviedo H, Romero G, Maccarone B, Essenfeld E, Convit A, Palacios LE (2015). Autologous tumor lysate/Bacillus Calmette-Guérin immunotherapy as an adjuvant to conventional breast cancer therapy. Clin Transl Oncol.

[16] Engelhardt JJ, Boldajipour B, Beemiller P, Pandurangi P, Sorensen C, Werb Z, Egeblad M, Krummel MF (2012). Marginating dendritic cells of the tumor microenvironment cross-present tumor antigens and stably engage tumor-specific T cells. Cancer Cell.

[17] Tran Janco JM, Lamichhane P, Karyampudi L, Knutson KL (2015). Tumor-infiltrating dendritic cells in cancer pathogenesis. J Immunol.

[18] Steinman RM, Hawiger D, Nussenzweig MC (2003). Tolerogenic dendritic cells. Annu Rev Immunol.

[19] Melief CJ (2003). Mini-review: regulation of cytotoxic T lymphocyte responses by dendritic cells: peaceful coexistence of cross-priming and direct priming?. Eur J Immunol.

[20] Steinman RM, Nussenzweig MC (2002). Avoiding horror autotoxicus: the importance of dendritic cells in peripheral T cell tolerance. Proc Natl Acad Sci U S A.

[21] Tang M, Diao J, Cattral MS (2017). Molecular mechanisms involved in dendritic cell dysfunction in cancer. Cell Mol Life Sci.

[22] Höpken UE, Lehmann I, Droese J, Lipp M, Schüler T, Rehm A (2005). The ratio between dendritic cells and T cells determines the outcome of their encounter: proliferation versus deletion. Eur J Immunol.

[23] Ruffell B, Au A, Rugo HS, Esserman LJ, Hwang ES, Coussens LM (2012). Leukocyte composition of human breast cancer. Proc Natl Acad Sci U S A.

[24] Li Q, Lao X, Pan Q, Ning N, Yet J, Xu Y, Li S, Chang AE (2011). Adoptive transfer of tumor reactive B cells confers host T-cell immunity and tumor regression. Clin Cancer Res.

[25] Zhang Y, Morgan R, Chen C, Cai Y, Clark E, Khan WN, Shin SU, Cho HM, Al Bayati A, Pimentel A, Rosenblatt JD (2016). Mammary-tumor-educated B cells acquire LAP/TGF-β and PD-L1 expression and suppress anti-tumor immune responses. Int Immunol.

[26] Tadmor T, Zhang Y, Cho HM, Podack ER, Rosenblatt JD (2011). The absence of B lymphocytes reduces the number and function of T regulatory cells and enhances the anti-tumor response in a murine tumor model. Cancer Immunol Immunother.

[27] Olkhanud PB, Damdinsuren B, Bodogai M, Gress RE, Sen R, Wejksza K, Malchinkhuu E, Wersto RP, Biragyn A (2011). Tumor-evoked regulatory B cells promote breast cancer metastasis by converting resting CD4^+^ T cells to T-regulatory cells. Cancer Res.

[28] Lim KH, Telisinghe PU, Abdullah MS, Ramasamy R (2010). Possible significance of differences in proportions of cytotoxic T cells and B-lineage cells in the tumour-infiltrating lymphocytes of typical and atypical medullary carcinomas of the breast. Cancer Immun.

[29] Janssen EM, Lemmens EE, Wolfe T, Christen U, von Herrath MG, Schoenberger SP (2003). CD4+ T cells are required for secondary expansion and memory in CD8+ T lymphocytes. Nature.

[30] Huang Y, Ma C, Zhang Q, Ye J, Wang F, Zhang Y, Hunborg P, Varvares MA, Hoft DF, Hsueh EC, Peng G (2015). CD4+ and CD8+ T cells have opposing roles in breast cancer progression and outcome. Oncotarget.

[31] Syed Khaja AS, Toor SM, El Salhat H, Faour I, Ul Haq N, Ali BR, Elkord E (2017). Preferential accumulation of regulatory T cells with highly immunosuppressive characteristics in breast tumor microenvironment. Oncotarget.

[32] Stanton SE, Disis ML (2016). Clinical signifcance of tumorinfltrating lymphocytes in breast cancer. J Immunother Cancer.

[33] Dushyanthen S, Beavis PA, Savas P, Teo ZL, Zhou C, Mansour M, Darcy PK, Loi S (2015). Relevance of tumorinfltrating lymphocytes in breast cancer. BMC Med.

[34] García-Martínez E, Gil GL, Benito AC, González-Billalabeitia E, Conesa MA, García García T, García-Garre E, Vicente V, Ayala de la Peña F (2014). Tumor-infiltrating immune cell profiles and their change after neoadjuvant chemotherapy predict response and prognosis of breast cancer. Breast Cancer Res.

[35] Pedroza-Pacheco I, Madrigal A, Saudemont A (2013). Interaction between natural killer cells and regulatory T cells: perspectives for immunotherapy. Cell Mol Immunol.

[36] Sonoda T, Sugimura K, Ikemoto S, Kawashima H, Nakatani T (2007). Significance of target cell infection and natural killer cells in the anti-tumor effects of bacillus Calmette-Guerin in murine bladder cancer. Oncol Rep.

[37] Ghiringhelli F, Ménard C, Terme M, Flament C, Taieb J, Chaput N, Puig PE, Novault S, Escudier B, Vivier E, Lecesne A, Robert C, Blay JY (2005). CD4+CD25+ regulatory T cells inhibit natural killer cell functions in a transforming growth factor-beta-dependent manner. J Exp Med.

[38] Wu JD (2004). Prevalent expression of the immunostimulatory MHC class I chain-related molecule is counteracted by shedding in prostate cancer. J Clin Invest.

[39] Cai L, Zhang Z, Zhou L, Wang H, Fu J, Zhang S, Shi M, Zhang H, Yang Y, Wu H, Tien P, Wang FS (2008). Functional impairment in circulating and intrahepatic NK cells and relative mechanism in hepatocellular carcinoma patients. Clin Immunol.

[40] Szebeni GJ, Vizler C, Nagy LI, Kitajka K, Puskas LG (2016). Pro-Tumoral Inflammatory Myeloid Cells as Emerging Therapeutic Targets. Int J Mol Sci.

[41] Gkogkou C, Frangia K, Saif MW, Trigidou R, Syrigos K (2014). Necrosis and apoptotic index as prognostic factors in non-small cell lung carcinoma: a review. Springerplus.

[42] Iqbal NT, Hussain R (2014). Non-specific immunity of BCG vaccine: A perspective of BCG immunotherapy. Trials Vaccinol.

[43] Abadie V, Badell E, Douillard P, Ensergueix D, Leenen PJ, Tanguy M, Fiette L, Saeland S, Gicquel B, Winter N (2005). Neutrophils rapidly migrate via lymphatics after Mycobacterium bovis BCG intradermal vaccination and shuttle live bacilli to the draining lymph nodes. Blood.

[44] Cicchelero L, de Rooster H, Sanders NN (2014). Various ways to improve whole cancer cell vaccines. Expert Rev Vaccines.

[45] Inoue H, Tani K (2014). Multimodal immunogenic cancer cell death as a consequence of anticancer cytotoxic treatments. Cell Death Differ.

[46] Ito T (2014). PAMPs and DAMPs as triggers for DIC. J Intensive Care.

[47] Kursunel MA, Esendagli G (2016). The untold story of IFN-γ in cancer biology. Cytokine Growth Factor Rev.

[48] Babjuk M, Burger M, Zigeuner R, Shariat SF, van Rhijn BW, Compérat E, Sylvester RJ, Kaasinen E, Böhle A, Palou Redorta J, Rouprêt M (2013). European Association of Urology. EAU guidelines on non-muscle-invasive urothelial carcinoma of the bladder: update 2013. Eur Urol.

[49] Shalapour S, Karin M (2015). Immunity, inflammation, and cancer: an eternal fight between good and evil. J Clin Invest.

[50] Goudin N, Chappert P, Mégret J, Gross DA, Rocha B, Azogui O (2016). Depletion of Regulatory T Cells Induces High Numbers of Dendritic Cells and Unmasks a Subset of Anti-Tumour CD8+CD11c+ PD-1lo Effector T Cells. PLoS One.

[51] Krempski J, Karyampudi L, Behrens MD, Erskine CL, Hartmann L, Dong H, Goode EL, Kalli KR, Knutson KL (2011). Tumor-infiltrating programmed death receptor-1+ dendritic cells mediate immune suppression in ovarian cancer. J Immunol.

[52] Scarlett UK, Rutkowski MR, Rauwerdink AM, Fields J, Escovar-Fadul X, Baird J, Cubillos-Ruiz JR, Jacobs AC, Gonzalez JL, Weaver J, Fiering S, Conejo-Garcia JR (2012). Ovarian cancer progression is controlled by phenotypic changes in dendritic cells. J Exp Med.

[53] Kocián P, Šedivcová M, Drgáč J, Cerná K, Hoch J, Kodet R, Bartůňková J, Špíšek R, Fialová A (2011). Tumorinfiltrating lymphocytes and dendritic cells in human colorectal cancer: their relationship to KRAS mutational status and disease recurrence. Hum Immunol.

[54] Chen W, Liang X, Peterson AJ, Munn DH, Blazar BR (2008). The indoleamine 2,3-dioxygenase pathway is essential for human plasmacytoid dendritic cell-induced adaptive T regulatory cell generation. J Immunol.

[55] Sisirak V, Faget J, Gobert M, Goutagny N, Vey N, Treilleux I, Renaudineau S, Poyet G, Labidi-Galy SI, Goddard-Leon S, Durand I, Le Mercier I, Bajard A (2012). Impaired IFN-α production by plasmacytoid dendritic cells favors regulatory T-cell expansion that may contribute to breast cancer progression. Cancer Res.

[56] Bauer CA, Kim EY, Marangoni F, Carrizosa E, Claudio NM, Mempel TR (2014). Dynamic Treg interactions with intratumoral APCs promote local CTL dysfunction. J Clin Invest.

[57] Jang JE, Hajdu CH, Liot C, Miller G, Dustin ML, Bar-Sagi D (2017). Crosstalk between Regulatory T Cells and Tumor-Associated Dendritic Cells Negates Anti-tumor Immunity in Pancreatic Cancer. Cell Rep.

[58] Diederichsen AC, Hjelmborg J, Christensen PB, Zeuthen J, Fenger C (2003). Prognostic value of the CD4+/CD8+ ratio of tumour infiltrating lymphocytes in colorectal cancer and HLA-DR expression on tumour cells. Cancer Immunol Immunother.

[59] Giuntoli RN, Webb TJ, Zoso A, Rogers O, Diaz-Montes TP, Bristow RE, Oelke M (2009). Ovarian cancer-associated ascites demonstrates altered immune environment: implications for antitumor immunity. Anticancer Res.

[60] Saber CN, Gronhoj LC, Dalianis T, von Buchwald C (2016). Immune cells and prognosis in HPV-associated oropharyngeal squamous cell carcinomas: review of the literature. Oral Oncol.

[61] Alonso-Alvarez S, Vidriales MB, Caballero MD, Blanco O, Puig N, Martin A, Peñarrubia MJ, Zato E, Galende J, Bárez A, Alcoceba M, Orfão A, González M (2017). The number of tumor infiltrating T-cell subsets in lymph nodes from patients with Hodgkin lymphoma is associated with the outcome after first line ABVD therapy. Leuk Lymphoma.

[62] Brandau S, Riemensberger J, Jacobsen M, Kemp D, Zhao W, Zhao X, Jocham D, Ratliff TL, Böhle A (2001). NK cells are essential for effective BCG immunotherapy. Int J Cancer.

[63] Dhiman R, Periasamy S, Barnes PF, Jaiswal AG, Paidipally P, Barnes AB, Tvinnereim A, Vankayalapati R (2012). NK1.1+ cells and IL-22 regulate vaccine-induced protective immunity against challenge with Mycobacterium tuberculosis. J Immunol.

[64] Crouse J, Xu HC, Lang PA, Oxenius A (2015). NK cells regulating T cell responses: mechanisms and outcome. Trends Immunol.

[65] Kroeger DR, Milne K, Nelson BH (2016). Tumor-Infiltrating Plasma Cells Are Associated with Tertiary Lymphoid Structures, Cytolytic T-Cell Responses, and Superior Prognosis in Ovarian Cancer. Clin Cancer Res.

[66] Fremd C, Schuetz F, Sohn C, Beckhove P, Domschke C (2013). B cell-regulated immune responses in tumor models and cancer patients. Oncoimmunology.

[67] Berntsson J, Nodin B, Eberhard J, Micke P, Jirström K (2016). Prognostic impact of tumour-infiltrating B cells and plasma cells in colorectal cancer. Int J Cancer.

[68] Duluc D, Corvaisier M, Blanchard S, Catala L, Descamps P, Gamelin E, Ponsoda S, Delneste Y, Hebbar M, Jeannin P (2009). Interferon-gamma reverses the immunosuppressive and protumoral properties and prevents the generation of human tumor-associated macrophages. Int J Cancer.

[69] Kumar V, Patel S, Tcyganov E, Gabrilovich DI (2016). The Nature of Myeloid-Derived Suppressor Cells in the Tumor Microenvironment. Trends Immunol.

[70] Doz E, Lombard R, Carreras F, Buzoni-Gatel D, Winter N (2013). Mycobacteria-infected dendritic cells attract neutrophils that produce IL-10 and specifically shut down Th17 CD4 T cells through their IL-10 receptor. J Immunol.

[71] Moghaddam A, Olszewska W, Wang B, Tregoning JS, Helson R, Sattentau QJ, Openshaw PJ (2006). A potential molecular mechanism for hypersensitivity caused by formalin-inactivated vaccines. Nat Med.

[72] Fan YC, Chiu HC, Chen LK, Chang GJ, Chiou SS (2015). Formalin Inactivation of Japanese Encephalitis Virus Vaccine Alters the Antigenicity and Immunogenicity of a Neutralization Epitope in Envelope Protein Domain III. PLoS Negl Trop Dis.

[73] Geijtenbeek TB, Gringhuis SI (2009). Signalling through C-type lectin receptors: shaping immune responses. Nat Rev Immunol.

[74] Madan-Lala R, Sia JK, King R, Adekambi T, Monin L, Khader SA, Pulendran B, Rengarajan J (2014). Mycobacterium tuberculosis impairs dendritic cell functions through the serine hydrolase Hip1. J Immunol.

[75] Feldman J, Goldwasser R, Mark S, Schwartz J, Orion I (2009). A mathematical model for tumour volume evaluation using two-dimensions. J Quant Appl Method.

[76] Mihara H, Boudaka A, Sugiyama T, Moriyama Y, Tominaga M (2011). Transient receptor potential vanilloid 4 (TRPV4)-dependent calcium influx and ATP release in mouse oesophageal keratinocytes. J Physiol.

[77] Iwamoto M, Allen RD (2004). Uptake and rapid transfer of fluorescent ceramide analogues to acidosomes (late endosomes) in Paramecium. J Histochem Cytochem.

[78] Tzekov R, Quezada A, Gautier M, Biggins D, Frances C, Mouzon B, Jamison J, Mullan M, Crawford F (2014). Repetitive mild traumatic brain injury causes optic nerve and retinal damage in a mouse model. J Neuropathol Exp Neurol.

[79] Pirog EC, Czerwinski W (1992). Diagnostic and prognostic significance of the mitotic index in endometrial adenocarcinoma. Gynecol Oncol.

[80] Moffitt P (1994). A methyl green-pyronin technique for demonstrating cell death in the murine tumour S180. Cell Biol Int.

[81] Mote RF, Muhm RL, Gigstad DC (1975). A staining method using acridine orange and auramine O for fungi and mycobacteria in bovine tissue. Stain Technol.

