# Supplementary material for: Autologous tumor cells/bacillus Calmette-Guérin/formalin-based novel breast cancer vaccine induces an immune antitumor response
Source: Oncotarget. 2018 Apr 17;9(29):20222–38. doi: 10.18632/oncotarget.25044 (PMC5945537; doi:10.18632/oncotarget.25044)
Supplement: Supplementary file 1 [file oncotarget-09-20222-s001.pdf]

Autologous tumor cells/bacillus Calmette-Guérin/formalin-based novel breast cancer vaccine induces an immune antitumor response

SUPPLEMENTARY MATERIALS

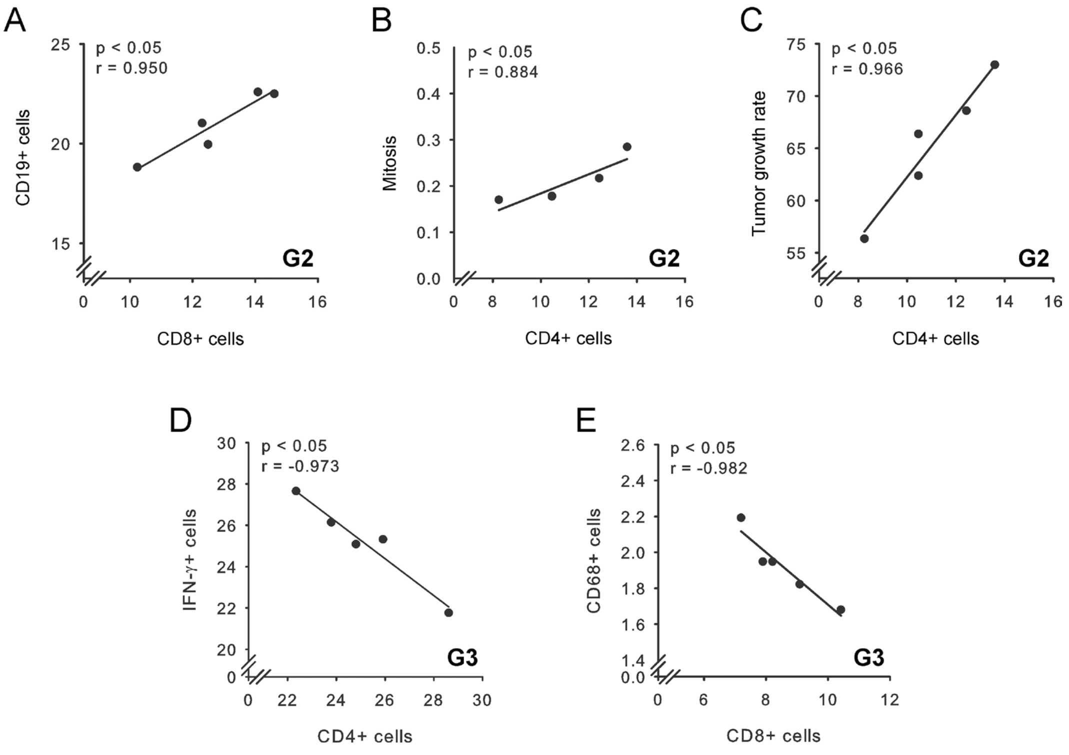

Supplementary Figure 1: Correlations calculated in tumor samples. (**A**) Correlation between $CD19^+$ and $CD8^+$ T cells in G2 was calculated to identify a possible $CD8^+$ T priming by B cells. (**B**) Correlation between $CD4^+$ T cells and mitosis percentage in G2. (**C**) Correlation between $CD4^+$ T cells and tumor growth rate in G2. (**D**) Correlation between $CD4^+$ T cells and IFN-γ^+ cells in G3. (**E**) Correlation between $CD68^+$ and $CD8^+$ T cells in G3. The symbols represent the media ± SEM value for the 5 mice in each specified group. The calculated value of the correlation is represented in each scatter diagram (*r*), and for all correlations the *p* value was lower than 0.05.
